# Occurrence, Formation from d-Fructose and 3-Deoxyglucosone, and Activity of the Carbohydrate-Derived β-Carbolines in Foods

**DOI:** 10.1021/acs.jafc.1c02281

**Published:** 2021-06-03

**Authors:** Tomás Herraiz, Fernando Vera

**Affiliations:** Instituto de Ciencia y Tecnología de Alimentos y Nutrición (ICTAN-CSIC), Spanish National Research Council (CSIC), Juan de la Cierva 3, 28006 Madrid, Spain

**Keywords:** carbohydrate-derived β-carbolines, alkaloids, tryptophan, 3-deoxyglucosone, Maillard reaction, advanced glycation

## Abstract

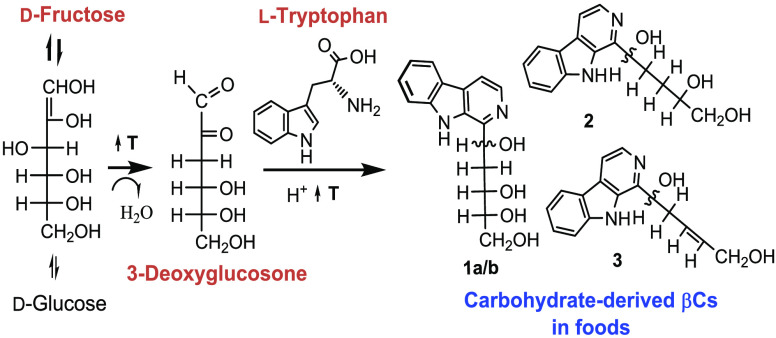

β-Carbolines are naturally occurring bioactive alkaloids. In this work, carbohydrate-derived β-carbolines (βCs), 1-(1,3,4,5-tetrahydroxypent-1-yl)-β-carboline isomers (**1a/b**), 1-(1,4,5-trihydroxypent-1-yl)-β-carboline (**2**), 1-(1,5-dihydroxypent-3-en-1-yl)-β-carboline (**3**), and 1-(1,2,3,4,5-pentahydroxypent-1-yl)-β-carboline (**4**) were identified and analyzed in commercial foods. The concentrations of βCs **1–4** in foods ranged from undetectable to 11.4 μg/g levels, suggesting their intake in the diet. Processed foods contained higher amounts than fresh or unprocessed foods, and the highest content was found in processed tomato and fruit products, sauces, and baked foods. βCs **1–3** were formed in foods during heating, and **1a/b** were the main compounds. The formation of carbohydrate-derived βCs was studied in model reactions of tryptophan and carbohydrates. They formed in reactions of tryptophan with glucose under acidic conditions at temperatures higher than 80 °C. The formation of **1a/b** was favored, but **2–3** increased at high temperatures. Noticeably, the βCs **1–3** formed in the reactions of tryptophan with fructose or sucrose, and the formation from fructose was much higher than from glucose. Thus, fructose was the main carbohydrate involved in the formation of **1–3**, whereas sucrose gave these βCs after acid hydrolysis. It is shown for the first time that the mechanism of formation of βCs **1–3** occurs from the sugar intermediate 3-deoxyglucosone that reacts with tryptophan affording these carbohydrate-derived βCs. A mechanism of reaction to give βCs **1–3** is proposed that relies on the tautomerism (keto–enediol or enamine–imine) of intermediates involved in the reaction. Carbohydrate βCs **1–4** were assessed as inhibitors of monoamine oxidase (MAO), as antioxidants, and for their interaction with DNA. They were not good inhibitors of MAO-A or -B, were poor antioxidants, and did not appreciably interact with DNA.

## Introduction

β-Carbolines (9*H*-pyrido[3,4-*b*]indole) (βCs) are indole alkaloids occurring in foods, plants, and biological systems.^1,2^ These alkaloids are bioactive compounds that exhibit an array of biological, pharmacological, and toxicological activities.^1−3^ They act on the central nervous system (CNS) through interaction with serotonin uptake, benzodiazepine receptor and imidazoline binding sites, and inhibit monoamine oxidase (MAO) and kinase enzymes.^3^ The βCs norharman and harman occurring in foods and cigarette smoke are potent inhibitors of MAO.^4,5^ These compounds exert antidepressant and behavioral effects owing to their effects on neurotransmitters and inhibition of MAO, and have been involved in drug and alcohol addictions.^6−8^ Some βCs are neuroprotectants or are involved in neurogenesis,^9^ while others can be bioactivated by N-methylation affording endogenous neurotoxins (β-carbolinium cations) analogues to 1-methyl-4-phenyl-1,2,3,6-tetrahydropyridine (MPTP) neurotoxin.^3^ In addition, some βCs are co-mutagenic, bind to DNA, and react with hydroxyl radical (OH^•^).^10,11^

**Figure 1:**
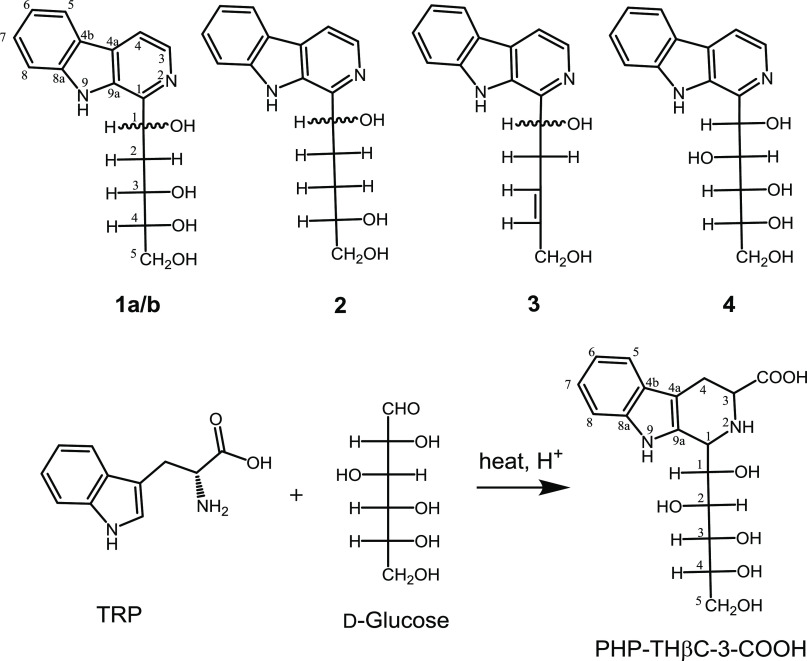
Chemical structures of carbohydrate-derived βCs **1-4** and the reaction of tryptophan with glucose to give 1-(1,2,3,4,5-pentahydroxypent-1-yl)-1,2,3,4-tetrahydro-β-carboline-3-carboxylic acid (PHP-THβC-3-COOH). The compounds are: 1-(1,3,4,5-tetrahydroxypent-1-yl)-β-carboline isomers (**1a/b)**, 1-(1,4,5-trihydroxypent-1-yl)-β-carboline (**2**), 1-(1,5-dihydroxypent-3-en-1-yl)-β-carboline (**3**), and 1-(1,2,3,4,5-pentahydroxypent-1-yl)-β-carboline (**4)**.

Depending on the pyridoindole ring oxidation, there are tetrahydro-β-carbolines (THβCs) and aromatic β-carbolines (βCs). THβCs are produced through a Pictet–Spengler reaction from indole-ethylamines or indole-ethylamino acids and aldehydes or α-keto acids. Tetrahydro-β-carboline-3-carboxylic acids (THβC-3-COOHs) arise from a reaction between l-tryptophan and aldehydes. These reactions occur in foods, and a number of THβCs including carbohydrate-derived THβCs have been identified and quantified in foods.^1,12^ Aromatic βCs arise from the oxidation of THβCs.^13^ Among them, the βCs norharman and harman are two main compounds that occur in foods and which are generated in meats and fish during cooking.^13,14^ These βCs arise from the corresponding THβC-3-COOHs that are the most abundant THβCs in foods.^15^ In addition, other aromatic βCs such as those derived from carbohydrates may appear in foods (Figure 1).^1,16−19^ Thus, glucose-derived aromatic βCs have been found in a number of foods and human urine.^1,17,20,21^ Some of these βCs have been also recently found in fruits of *Nitraria tangutorum*.^22^ However, a few reports exist so far on the occurrence and activity of these compounds, whereas their mechanism of formation remains unknown. In this regard, carbohydrate-derived THβCs appear in reactions of tryptophan with carbohydrates.^12,23,24^ It has been proposed that the carbohydrate-derived aromatic βCs might arise from the corresponding pentahydroxypentyl-THβC-3-COOH (PHP-THβC-3-COOH) formed from glucose and tryptophan (Figure 1).^1,12,16,23^ In fact, the compound PHP-THβC-3-COOH has been previously reported in foods and relatively high amounts found in tomato products, fruit juices, and jams,^12^ and also found in human urine.^25,26^

As the βCs exhibit bioactive and/or toxic actions and they may appear in tissues and biological fluids, the exposure to these compounds in the diet is a matter of interest. The purpose of this work was to study the occurrence of carbohydrate-derived βCs in a wide range of commercial foods and, subsequently, investigate their mechanisms of formation highlighting the precursors and intermediates involved. Finally, the study assesses the activity of carbohydrate-derived βCs identified in foods as MAO inhibitors, antioxidants, and their interaction/intercalation with DNA.

## Materials and Methods

### Chemical Compounds and Foods

Commercial samples of foods were purchased from local supermarkets to cover a wide range of foods and used to analyze carbohydrate-derived β-carbolines. d-(+)-Glucose monohydrate, lactose, and maltose were obtained from Merck, d-(-)-fructose from Sigma, d-(+)-sucrose from Scharlau, and 3-deoxy-d-glucosone from Biosynth-Carbosynth. l-Tryptophan, tryptamine, l-tryptophan methyl ester, harman, and norharman were purchased from Sigma. 1-(1,2,3,4,5-Pentahydroxypent-1-yl)-1,2,3,4-tetrahydro-β-carboline-3-carboxylic acid (PHP-THβC-3-COOH) was obtained from l-tryptophan and d-glucose as previously.^12^ The carbohydrate-derived β-carbolines, 1-(1,3,4,5-tetrahydroxypent-1-yl)-β-carboline diastereoisomers (**1a/b**), 1-(1,4,5-trihydroxypent-1-yl)-β-carboline (**2**), and 1-(1,5-dihydroxypent-3-en-1-yl)-β-carboline (**3**) were obtained from a reaction of glucose with tryptophan heated in pH 1 and isolated with C18 column chromatography, as previously,^17^ and 1-(1,2,3,4,5-pentahydroxypent-1-yl)-β-carboline (**4**) obtained following oxidation of PHP-THβC-3-COOH^23^ (Figure 1). The spectral data of these βCs have been reported.^12,17,20,22,23^ The purity of βCs was higher than 90% by high-performance liquid chromatography (HPLC). 1-Ethyl-β-carboline (EβC) was obtained from the chemical oxidation of 1-ethyl-1,2,3,4-tetrahydro-β-carboline-3-carboxylic acid (ETCA) with sodium dichromate and solvent extraction.^13^ Monoamine oxidase (MAO) enzymes were obtained from Corning (Gentest), horseradish peroxidase (HRP), calf thymus DNA (type I highly polymerized), ethidium bromide, ascorbic acid, catechin, quercetin, and 3,3′,5,5′-tetramethylbenzidine (TMB) from Sigma-Aldrich, and H_2_O_2_ from Scharlau.

### Isolation of Carbohydrate-Derived β-Carbolines by Solid-Phase Extraction (SPE)

Samples of solid foods (4–7 g) were added with 0.6 M HClO_4_ (15–20 mL), homogenized using an ultraturrax homogenizer, and subsequently centrifuged at 10000 rpm for 15 min at 0–5 °C. Liquid samples of foods (juices, beverages, vinegars, teas) or diluted samples (e.g., soy sauce) were centrifuged and acidified with HCl 0.1 M. The isolation of carbohydrate βC was carried out by solid-phase extraction (SPE) using propylsulfonic acid-derivatized silica PRS columns (Bond Elut, 500 mg, 3 mL size, Varian, Harbor City, CA) as previously with minor modifications.^14^ The procedure of isolation was optimized for the recovery of carbohydrate βCs. The conditioning of PRS columns was made with methanol and 0.1 M HCl. Aliquots (5 mL) were spiked with 125 μL of 1-ethyl-β-carboline solution (EβC) (0.2 mg/L) as internal standard (IS) and subsequently loaded onto PRS columns using a vacuum manifold. After washing with deionized water (6 mL), the carbohydrate βCs were eluted with 3 mL of 0.4 M K_2_HPO_4_ (pH 9.1) followed with 3 mL of 0.4 M K_2_HPO_4_ (pH 9.1)/methanol (1:1). The eluates were joined and injected into HPLC and HPLC mass spectrometry (MS) columns. The evaluation of the performance and quantitative analysis were carried out as mentioned below.

### Formation of Carbohydrate-Derived βCs in Model Reactions and Foods

Studies using model reactions containing l-tryptophan and carbohydrates, both in low and high concentrations, were carried out to evaluate the effects of pH, temperature, and carbohydrate on the formation of the carbohydrate-derived βCs as follows: (a) two concentrations of tryptophan (0.5 or 2 g/L) and equimolar concentrations of glucose (5 or 40 g/L), fructose (4.54 or 36.6 g/L), or sucrose (8.5 or 69.1 g/L) in phosphate solutions (100 mM) adjusted to different pHs (1.3, 3.1, 5, 7.4, and 9) were placed in glass tubes with ground-glass stoppers and reacted in an oven for 20 h (80 or 90 °C). In addition, controls with only tryptophan or carbohydrates were also carried out. (b) Solutions of tryptophan (2 g/L) and glucose (40 g/L), fructose (36.6 g/L), or sucrose (69.1 g/L) in phosphate buffer pH 3.1 were reacted at different temperatures to cover a wide range (from 37 to 130 °C). (c) Reactions were also carried out with tryptophan (2 g/L) and maltose (72.7 g/L) or lactose (72.7 g/L) at pH 1.3 and 3.1 (90 °C, 20 h). On the other hand, tryptamine (2 g/L) or tryptophan methyl ester (2 g/L) instead of tryptophan were reacted with glucose (40 g/L) (pH 3.1, 3 h, 110 °C). An aliquot of the reactions was injected into the reversed-phase (RP)-HPLC, analyzed by a diode array detector (DAD) and fluorescence as mentioned below, and subjected to HPLC-MS for compound identification. On the other hand, to study the formation of carbohydrate βCs in processed foods, white grapes and tomatoes (cherry) were subjected to drying in an oven at 80 °C and the carbohydrate βCs were isolated by SPE as reported above and analyzed by RP-HPLC. The same analysis was done with the raw samples. To study the conversion of possible precursors into products, compounds PHP-THβC-3-COOH, **1**, and **4** (100-500 μM, pH 1–7) were heated in an oven (140 °C, 1.4–4 h, or 110 °C, 3 h) or microwave, reconstituted to the original volume, and injected into HPLC and HPLC-MS. On the other hand, PHP-THβC-3-COOH was also oxidized (2 mM H_2_O_2_, 2 h, 37 °C or 0.025 mg/mL HRP, 500 μM H_2_O_2_, 40 min, 37 °C) and injected into HPLC and HPLC-MS columns. Finally, to highlight the mechanism of formation, model reactions of 3-deoxyglucosone (0.1 mg/mL) and tryptophan (0.5 mg/mL) in phosphate solutions adjusted to pH 1.3 or 3.1 were carried out at 90 and 110 °C for 2–4 h, and the products formed were analyzed by RP-HPLC and identified by HPLC-MS. All of the reactions were carried out in duplicate.

### Chromatographic and Quantitative Analyses of Carbohydrate-Derived βCs in Foods and Model Reactions, and Identification by HPLC-MS

Chromatographic analysis of carbohydrate βCs was performed using a 1050 high-performance liquid chromatograph with a 1100 series DAD (Agilent) and a 1046A fluorescence detector controlled by a Chemstation (Agilent Technologies). A 150 mm × 3.9 mm, 5 μm, Novapak C18 column (Waters) was used for HPLC separation. Eluents were 50 mM ammonium phosphate buffer adjusted to pH 3 with phosphoric acid (eluent A) and 20% of eluent A in acetonitrile (eluent B). The gradient was 0% B to 32% B in 8 min, then 90% B at 18 min, and 100% B at 20 min. The flow rate was 1 mL/min, the oven temperature was 40 °C, and the injection volume was 20 μL.

The carbohydrate βCs isolated from foods by SPE were analyzed by HPLC with fluorescence detection at 300 nm of excitation and 433 nm of emission. Quantitative analysis was obtained using standard solutions of known concentration of **1** against EβC used as an internal standard (IS) that were carried out through the entire SPE isolation procedure. The use of IS allowed us to correct for possible matrix effects occurring during SPE. The standard solutions were made in an acidic media similar to those used in SPE of foods and covered a range of concentrations found in foods (25–1000 μg/L). A linear calibration curve (*r*^2^ = 0.99) of peak area of the βC **1** divided by IS area vs concentration of the compound was obtained and used for quantitation. The performance and validation of the method gave good repeatability (2.5% RSD, *n* = 4), accuracy (3.4% mean error, *n* = 4), and recovery (95.7%, *n* = 4) (compound **1**, 100 μg/L). The recoveries in the SPE after spiking the food samples with **1** (1000 μg/L) were 95.8, 97.2, 92.3, 85.2, and 97% (*n* = 2) for tomato puree, jam, bread, chocolate, and pineapple juice, respectively. The analysis of carbohydrate βCs in model reactions was carried out by HPLC with DAD and fluorescence detection. Quantitation was carried out with calibration curves of βC **1** response at 254 nm and fluorescence (300 nm excitation and 433 nm emission) against concentration (0.01–1 mM range). The concentration of PHP-THβC-3-COOH in model reactions was determined by HPLC with absorbance detection at 280 nm and calculated from a calibration curve of response against concentration of this compound (0.01–1 mM range).

For identification purposes, the chromatographic peaks of carbohydrate βCs **1-4** in foods and model reactions were co-injected with authentic standards, and the DAD and fluorescence spectra were obtained. Identification was confirmed by HPLC-MS. For that, SPE extracts from foods and aliquots of model reactions were analyzed by HPLC-MS. SPE fractions were concentrated using a vacuum concentrator and analyzed by HPLC-MS (electrospray ionization (ESI) mode). The HPLC-MS analysis was carried out with an apparatus HPLC-MS Agilent equipped with 1200 series quaternary pump and DAD coupled to a 6110 single-quadrupole MSD detector working in electrospray ionization mode (API-ESI). Chromatographic separation was accomplished with a 3.9 mm ×150 mm Novapak C18 column (Waters) with eluents 0.5% formic acid (A) and 0.5% formic acid in acetonitrile (B), and using a linear gradient from 0 to 100% B in 15 min, a flow rate of 0.6 mL/min, and a temperature of 40 °C. Mass spectra were acquired in ESI positive-ion ionization mode at various fragmentor voltages (90 and 150 V) with an acquisition mass range of 50–800 u. The conditions in the mass spectrometer were: gas temperature, 350 °C; drying gas flow, 12 L/min; nebulizing gas pressure, 35 psig; and capillary voltage, 3000 V.

### Activity of Carbohydrate-Derived βCs as Inhibitors of MAO, Antioxidants, and DNA-Interaction Agents

The activity of carbohydrate βCs **1–4** and norharman and harman as inhibitors of MAO enzymes was studied as previously.^27^ Briefly, protein fractions containing MAO-A or -B (Corning-Gentest) were diluted to the desired concentrations in 100 mM potassium phosphate buffer (pH 7.4). A 0.2 mL reaction mixture containing 0.01 mg/mL protein, carbohydrate βCs (**1-4**), norharman or harman (from 0 to 50 μM range), and 0.25 mM kynuramine in 100 mM potassium phosphate (pH 7.4) was incubated at 37 °C for 40 min. After incubation, the reaction was stopped by the addition of 2 N NaOH (75 μL), followed by the addition of 70% HClO_4_ (25 μL), and the sample was centrifuged (10 000*g*) for 10 min. The supernatant (20 μL) was injected into the HPLC, and the kynuramine deamination product 4-hydroxyquinoline was determined by RP-HPLC-diode array detector at 320 nm. The inhibitors clorgyline (MAO-A) and R-deprenyl (MAO-B) were used as positive control for inhibition. Incubations were carried out in duplicate, and IC_50_ values calculated using GraphPad Prism software.

The antioxidant activity of carbohydrate βC **1–4** was assessed by scavenging of the radical TMB^•+^ (blue color) that is eliminated in the presence of antioxidants.^28^ For that, the radical cation TMB (TMB^•+^) was obtained from TMB (250 μM), HRP (25 μg/mL), and H_2_O_2_ (100 μM) that reacted at room temperature in 100 mM buffer phosphate (pH 7.4) for 1 min. Subsequently, compounds **1–4** (30 μM) and the antioxidants ascorbic acid (30 μM), catechin (30 μM), or quercetin (30 μM) were added to the mixture containing TMB cation radical, and the elimination of the radical (decoloration) followed at 650 nm over time (10 min). The activity of compounds **1–4** was compared with that of antioxidants.

The interaction of compounds **1–4** with calf thymus DNA was studied by UV–vis spectroscopy and compared with ethidium bromide, a known intercalating agent.^29,30^ The interaction between DNA and a ligand molecule can be examined by studying the shifting of the position of the maximum and the intensity of the absorption bands from when the ligand is free in solution or when is bound with DNA.^31^ For that, solutions (final volume 500 μL) of carbohydrate βC **1–4** (from 25 to 50 μM), or ethidium bromide (25 μM) in 10 mM trisaminomethane (TRIS) buffer (pH 7.4) placed in a quartz cuvette were added with successive volumes of calf thymus DNA (0, 2.5, 5, 7.5, 10, and 10 μL corresponding to 0, 6, 18, 36.4, 61.7, and 85 μM DNA calculated with ε at 260 nm), mixed, and UV–vis spectra were acquired from 220 to 800 nm over time (after 25 min of each addition of DNA) in a spectrophotometer (T70+ UV–vis spectrophotometer PG Instruments).

## Results

### Identification and Occurrence of Carbohydrate βCs in Foods and Model Reactions

**Figure 2:**
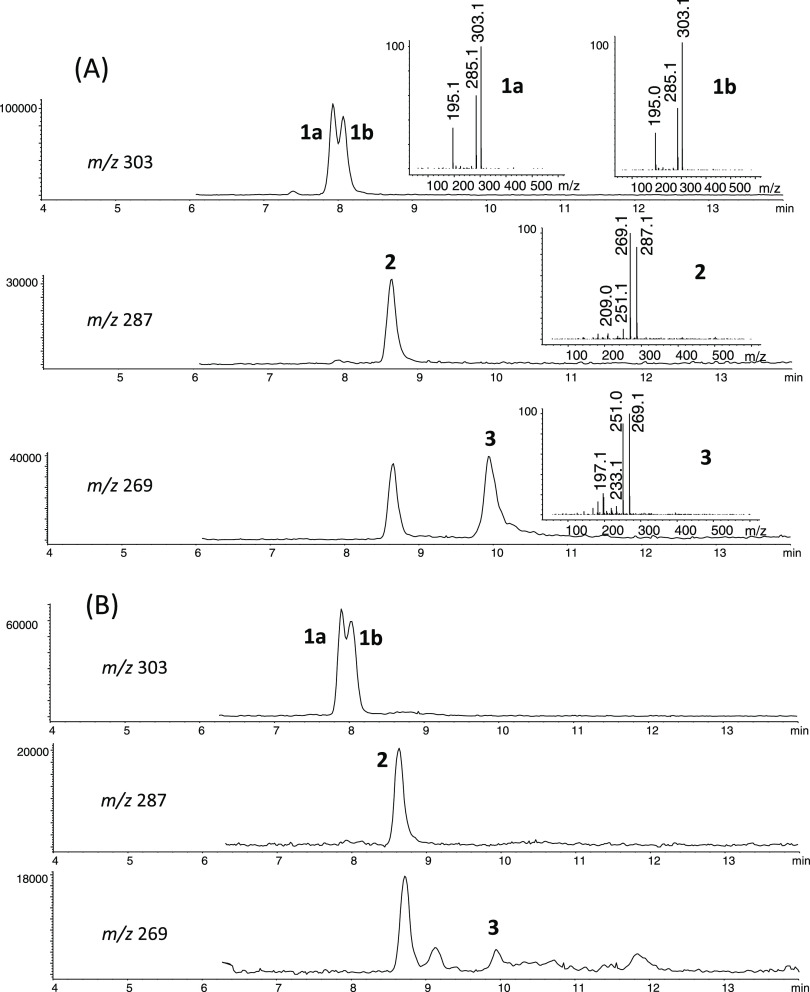
(A) HPLC-MS extracted ion chromatograms (EIC) and ESI-mass spectra (ionization voltage, 150 V) of the carbohydrate-derived βCs **1–3** in model reaction of tryptophan (2 g/L) and fructose (36.6 g/L) (90 °C, pH 3.1, 20 h). (B) HPLC-MS extracted ion chromatograms (ionization voltage, 150 V) of the carbohydrate-derived βCs **1–3** in concentrated tomato paste. The *m*/*z* ions are: 303 [M + H]^+^, 285 [303-18]^+^ and 195 [285-C_3_H_6_O_3_]^+^ in **1a/b**; 287 [M + H]^+^, 269 [287-18]^+^, 251 [269-18]^+^, and 209 [269-C_2_H_4_O_2_]^+^ in **2**; and 269 [M + H]^+^, 251 [269-18]^+^, and 197 [269-C_4_H_8_O]^+^ in **3**. Compounds are as in Figure 1.

**Figure 3:**
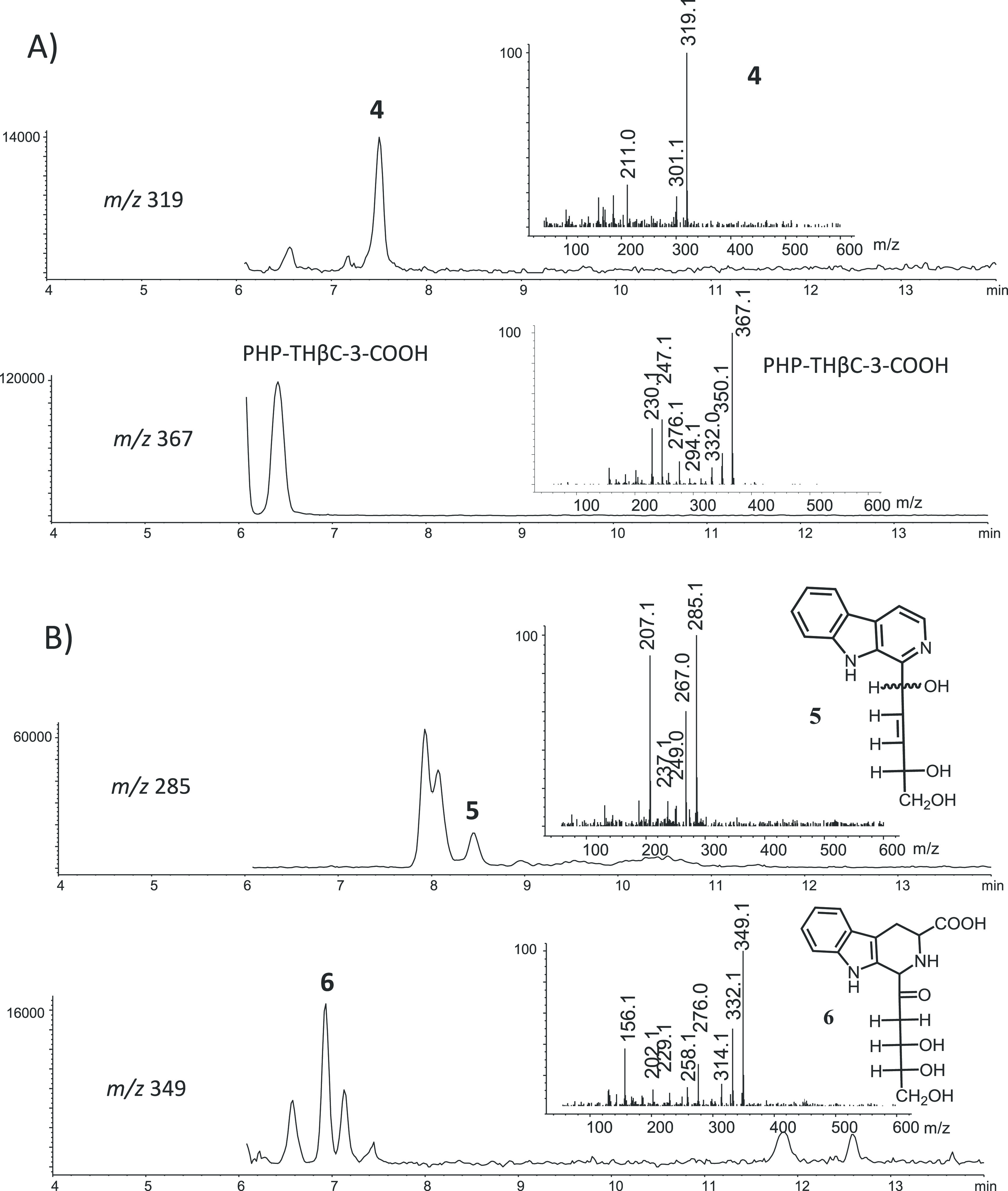
(A) HPLC-MS extracted ion chromatograms (EIC) and ESI-mass spectra (ionization voltage, 150 V) of the carbohydrate-derived βC **4** and PHP-THβC-3-COOH in model reactions of tryptophan (0.5 g/L) and glucose (5 g/L) (90 °C, pH 1.3, 20 h) (compounds are as in Figure 1). (B) HPLC-MS extracted ion chromatograms (EIC) and ESI-mass spectra (ionization voltage, 150 V) of compounds identified as (1,4,5-trihydroxypent-2-en-1-yl)-β-carboline (**5**) and 1-(1-keto-3,4,5-trihydroxypent-1-yl)-1,2,3,4-tetrahydro-β-carboline-3-carboxylic acid (**6**) in reactions of tryptophan (2 g/L) and fructose (36.6 g/L) (90 °C, pH 3.1, 20 h). The *m*/*z* ions are: 319 [M + H]^+^, 301 [319-18]^+^, and 211 [301-C_3_H_6_O_3_]^+^ in **4**; 367 [M + H]^+^, 350 [367-17]^+^, 332 [350-18]^+^, and 294 [367-73]^+^ in PHP-THβC-3-COOH; 285 [M + H]^+^, 267 [285-18]^+^, 207 [267-C_2_H_4_O_2_]^+^, 249 [267-18]^+^, and 237 [267-CH_2_O]^+^ in **5**; and 349 [M + H]^+^, 332 [349-17]^+^, 314 [332-18]^+^, and 276 [349-73]^+^ in **6**.

The carbohydrate-derived βCs **1–3** (Figure 1) occurring in foods and model reactions of tryptophan and carbohydrates were analyzed by HPLC and identified by UV–vis and fluorescence spectra as well as by coelution with authentic standards. Identification was confirmed by HPLC-MS (Figure 2). They were identified as 1-(1,3,4,5-tetrahydroxypent-1-yl)-β-carboline isomers (**1a/b)**, 1-(1,4,5-trihydroxypent-1-yl)-β-carboline (**2**), and 1-(1,5-dihydroxypent-3-en-1-yl)-β-carboline (**3**) in agreement with previous results.^17,20^ The compounds appeared in model reactions of tryptophan and glucose but also in the reactions of tryptophan and fructose (Figure 2). The mass spectra afforded the protonated molecular ion [M + H]^+^ and a fragmentation pattern dominated by successive loss of water (*m*/*z* 18) and fragmentation losses of C_3_H_6_O_3_ in **1**, C_2_H_4_O_2_ in **2**, and CH_2_O and C_4_H_8_O in **3**.^17^ The compounds have different C-1′ configurations that were separated by HPLC in the case of **1a/b** diastereoisomers. Besides **1–3**, the compounds 1-(1,2,3,4,5-pentahydroxypent-1-yl)-1,2,3,4-tetrahydro-β-carboline-3-carboxylic acid (PHP-THβC-3-COOH) and 1-(1,2,3,4,5-pentahydroxypent-1-yl)-β-carboline (**4**) were identified in foods and model reactions of tryptophan and glucose (Figures 1 and 3).^12^ Moreover, on the basis of the mass spectra and fragmentation pattern, two additional β-carbolines were identified as 1-(1,4,5-trihydroxypent-2-en-1-yl)-β-carboline (**5**) and 1-(1-keto-3,4,5-trihydroxypent-1-yl)-1,2,3,4-tetrahydro-β-carboline-3-carboxylic acid (**6**) (Figure 3) in model reactions of tryptophan and glucose or fructose, and in reactions of tryptophan and fructose, respectively. These compounds were collected from the HPLC column and afforded high-resolution mass spectra (ESI-Q-TOF-MS, Agilent) of *m*/*z* 285.1223 [M + H]^+^ (calculated for [C_16_H_16_N_2_0_3_+H]^+^, 285.1234) (**5**) and *m*/*z* 349.1370 [M + H]^+^ (calculated for [C_17_H_20_N_2_O_6_+H]^+^, 349.1394) (**6**).

**Figure 4:**
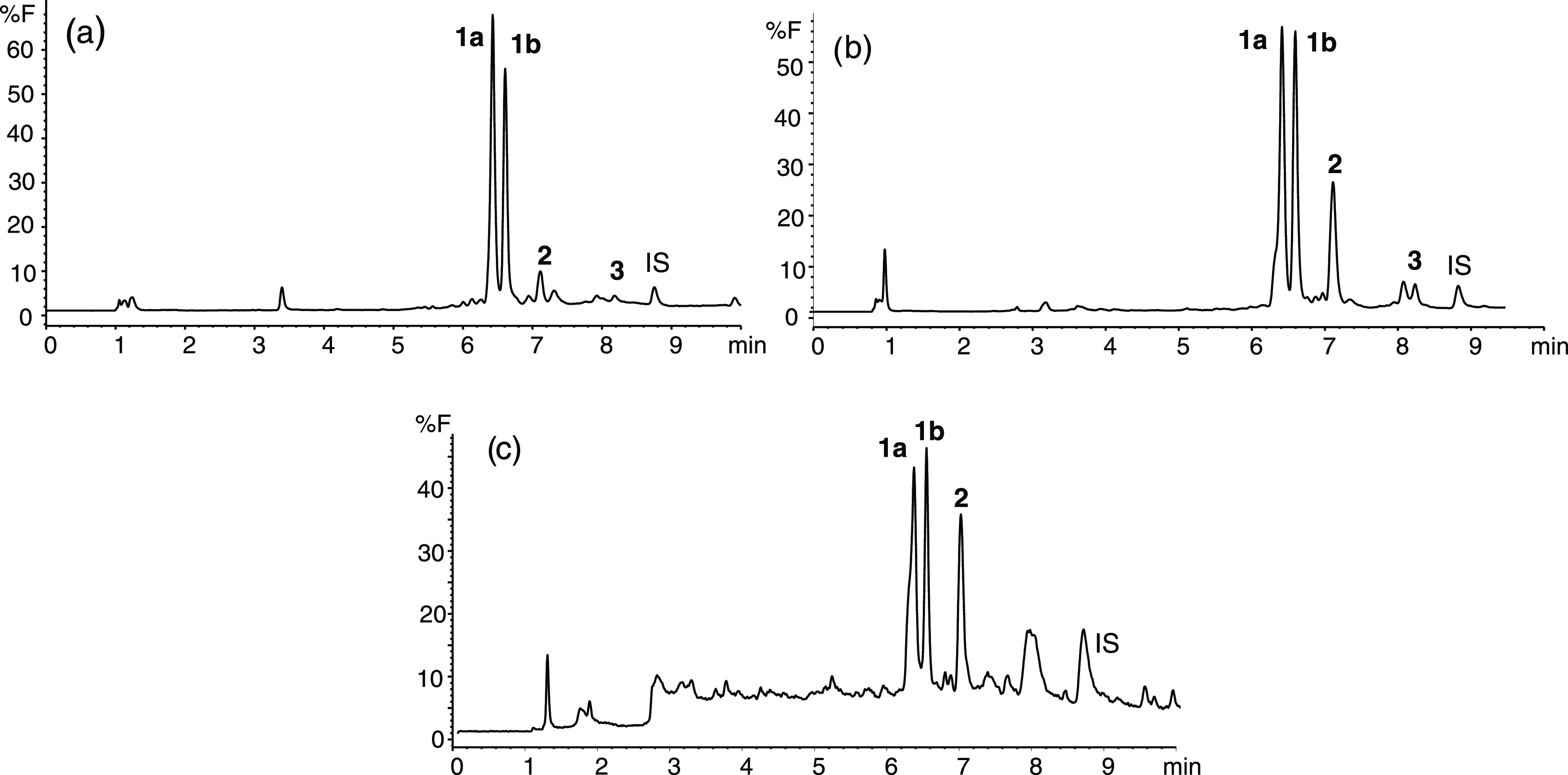
HPLC chromatograms of carbohydrate βCs isolated in tomato juice (a), raisins (b), and chocolate (c) obtained after SPE cleanup. Compounds are as in Figure 1.

The carbohydrate βCs **1–3** were analyzed in commercial foods by HPLC-fluorescence after SPE (examples are shown in Figure 4). Their content ranged from undetected to several μg/g or mg/L levels, with **1a/b** being the major compounds (Table 1). These β-carbolines appeared in the highest content in processed tomato products, including tomato juice, fried tomato sauce, ketchup, tomato concentrate, and dried tomato. Low processed samples such as natural tomato puree (from crushed tomatoes) did not contain or contained a very low level of carbohydrate βCs, and tomato juice elaborated from concentrate juice contained higher amounts than juices made not from concentrate. They appeared in relatively high amounts in processed fruit products such as dried fruits, jams and juices with those from pineapple, pineapple and grape or tropical juice containing the highest levels, whereas other juices such as orange or apple juices contained low amounts. The carbohydrate βCs were present in sauces including soy sauce. They were also present in baked foods such as bread and breakfast cereals and bars, biscuits, and cookies. In contrast, flours from wheat, corn, and rice did not have carbohydrate βCs. These compounds were present in chocolate, whereas very low levels were found in coffee or tea. Most nuts contained very low amounts of **1–3**. In contrast, carbohydrate βCs **1–3** appeared in dehydrated fruits, particularly in raisins and tomatoes. A number of foods did not contain or contained very low amounts or traces of carbohydrate βCs **1–3**. Those include fresh, smoked or cooked meat or fish, or dairy products such as milk, cheese, or yogurt. They were not found in sausages with exception of some cured sausages. Neither, they occurred in alcoholic beverages such as wines, liquors, or distilled beverages and trace levels appeared in beer. The compounds were not found in vinegars made from wine or cider, but balsamic vinegar that is made from heated must was an exception. The proportion of **1–3** in foods varied greatly, but usually **1a/b** were the main carbohydrate βCs. Nevertheless, relatively high levels of **2** and **3** appeared in some samples of baked foods and breads. The presence of βCs **4** and **5** was studied in foods, but they only appeared in a few samples, and the content of **4** ranged from undetectable in most samples to less than 0.2 μg/g in samples of tomato products (juices, sauces, fried, and dried tomato) and dried grapes.

**Table 1:** Concentration of Carbohydrate βCs (μg/L^1^ or μg/g^2^) Determined in Foods^a^

	**1a**			**1b**			**2**			**3**		
**food samples**	**mean**	**SD**	**range**	**mean**	**SD**	**range**	**mean**	**SD**	**range**	**mean**	**SD**	**range**
tomato juice^1^ (*n* = 11)	1494	1019	15–3575	1365	914.5	12.2–3097	274.3	230.4	5.1–797.8	54.3	53.6	nd–123.1
tomato juice (concentrate)^1^ (*n* = 8)	1918	841.4	971–3575	1762	715.5	1020–3097	352.0	222.9	150.6–798	72.4	52.0	nd–123.1
tomato juice (not concentrate)^1^ (*n* = 3)	365.9	304.3	15–575	305.1	269.7	12.2–543.2	67.18	54.31	5.1–105.9	6.1	10.6	nd–18.4
pineapple juice^1^ (*n* = 14)	111.3	113.7	nd–308.2	102.1	111.3	nd–319	16.9	23.4	nd–85.1	2.1	8.3	nd–32.3
tropical juice^1^ (*n* = 4)	142.1	113.9	49.3–304	154.0	122.3	46.4–317.3	26.04	9.846	15.05–34.35	43.13	86.25	nd–172.5
pineapple + grape juice^1^ (*n* = 8)	158.9	135.0	9.6–363.5	154.3	100.8	9.5–294.4	34.83	38.43	nd–93.2	3.9	4.8	nd–12.3
orange juice^1^ (*n* = 6)	28.5	23.2	nd–78	32.6	34.7	nd–110.9	13.7	21.64	nd–62.8	18.15	16.19	nd–49.8
juice (apple, peach, grape)^1^ (*n* = 15)	29.8	37.6	nd–103	36.4	45.7	nd–132.9	18.98	18.9	nd–50.7	nd		
fresh vegetable beverage/cream^2^ (*n* = 7)	0.027	0.024	nd–0.056	0.027	0.022	nd–0.051	0.003	0.007	nd–0.02	0.002	0.004	nd–0.01
jam^2^ (*n* = 11)	0.044	0.077	0.002–0.27	0.037	0.055	0.002–0.19	0.013	0.012	nd–0.034	0.005	0.010	nd–0.030
fried tomato sauce^2^ (*n* = 9)	1.78	1.44	0.44–4.97	1.57	1.34	0.42–4.6	0.28	0.17	0.054–0.61	0.054	0.059	nd–0.15
natural tomato puree^2^ (*n* = 6)	0.003	0.007	nd–0.018	0.002	0.005	nd–0.013	0.002	0.005	nd–0.011	0.002	0.002	nd–0.009
tomato puree conc.^2^ (*n* = 1)	7.6			6.4			1.6			0.49		
ketchup^2^ (*n* = 8)	1.43	1.38	0.24–4.3	1.28	1.19	0.24–3.8	0.26	0.22	0.024–0.71	0.070	0.074	nd–0.21
sauce^2^ (*n* = 8)	0.63	0.47	0.2–1.55	0.65	0.48	0.2–1.6	0.22	0.24	nd–0.7	0.038	0.06	nd–0.16
soy sauce^1^ (*n* = 3)	954.6	236.1	713.2–1185	1050	118.1	965.6–1185	968.8	104.2	803–1088			
wine vinegar^1^ (*n* = 4)	nd			nd			nd			nd		
cider vinegar^1^ (*n* = 2)	nd			nd			nd			nd		
vinegar balsamic^1^ (*n* = 1)	319.5			385.0			94.3			nd		
fried/caramelized onion^2^ (*n* = 3)	0.19	0.18	0.044–0.39	0.18	0.19	0.031–0.40	0.12	0.205	nd–0.36	nd		
dried tomato^2^ (*n* = 2)	2.35	3.0	0.22–4.48	2.26	2.97	0.15–4.4	0.97	1.33	0.029–1.91	0.34	0.37	0.08–0.60
dried fruit^2^ (*n* = 8)	0.11	0.10	nd–0.26	0.10	0.11	nd–0.26	0.033	0.05	nd–0.14	0.004	0.012	nd–0.035
raisin (dried grape)^2^ (*n* = 12)	0.78	0.78	0.07–2.85	0.79	0.63	0.10−1.93	0.54	0.44	nd–1.46	0.026	0.040	nd–0.12
nut^2^ (*n* = 6)	0.02	0.017	nd–0.05	0.025	0.019	nd–0.05	0.06	0.04	0.025–0.13	nd		
french fries^2^ (*n* = 2)	nd			nd			nd			nd		
chocolate^2^ (*n* = 7)	0.24	0.15	0.13–0.58	0.265	0.19	0.008–0.68	0.26	0.18	0.09–0.59	0.03	0.053	nd–0.135
breakfast cereal/bar^2^ (*n* = 9)	0.23	0.25	0.01–0.64	0.25	0.273	0.008–0.71	0.17	0.23	nd–0.79	0.028	0.055	nd–0.16
bread^2^ (*n* = 3)	0.04	0.03	0.01–0.07	0.05	0.03	0.012–0.077	0.31	0.086	0.21–0.38	0.017	0.003	nd–0.03
bread toasted^2^ (*n* = 3)	0.30	0.12	0.19–0.43	0.31	0.15	0.18–0.47	1.15	0.35	0.76–1.46	0.17	0.025	0.14–0.19
cookies/baked good^2^ (*n* = 8)	0.05	0.065	nd–0.178	0.06	0.063	nd–0.167	0.06	0.084	nd–0.26	0.007	0.006	nd–0.013
flour (wheat, corn, rice)^2^ (*n* = 4)	nd			nd			nd			nd		
tea^1^ (*n* = 4)	1.7	1.2	nd–2.7	1.8	1.2	nd–2.7	1.03	1.28	nd–2.6	nd		
coffee^1^ (*n* = 12)	8.7	10.7	nd–25.4	6.6	9.3	nd–22	nd			nd		
milk^1^ (*n* = 4)	nd			nd			nd			nd		
yogurt^2^ (*n* = 3)	nd			nd			nd			nd		
cheese^2^ (*n* = 5)	nd			nd			nd			nd		
cooked meat (pork/beef)^2^ (*n* = 4)	nd			nd			nd			nd		
cooked fish^2^ (*n* = 3)	nd			nd			nd			nd		
smoked fish^2^ (*n* = 4)	nd			nd			nd			nd		
sausage^2^ (*n* = 4)	nd			nd			nd			nd		
cured sausage^2^ (*n* = 2)	0.04	0.008	0.03–0.05	0.06	0.014	0.054–0.074	0.02	0.01	0.017–0.031	nd		
wine^1^ (*n* = 6)	nd			nd			nd			nd		
beer^1^ (*n* = 5)	29.2	40	nd–98	31.4	45	nd–108	5.3	9	nd–20.5	nd		
alcoholic beverages/liqueur^1^ (*n* = 6)	nd			nd			nd			nd		

a nd: not detected.

### Insights into the Mechanism of Formation of Carbohydrate βCs

**Figure 5:**
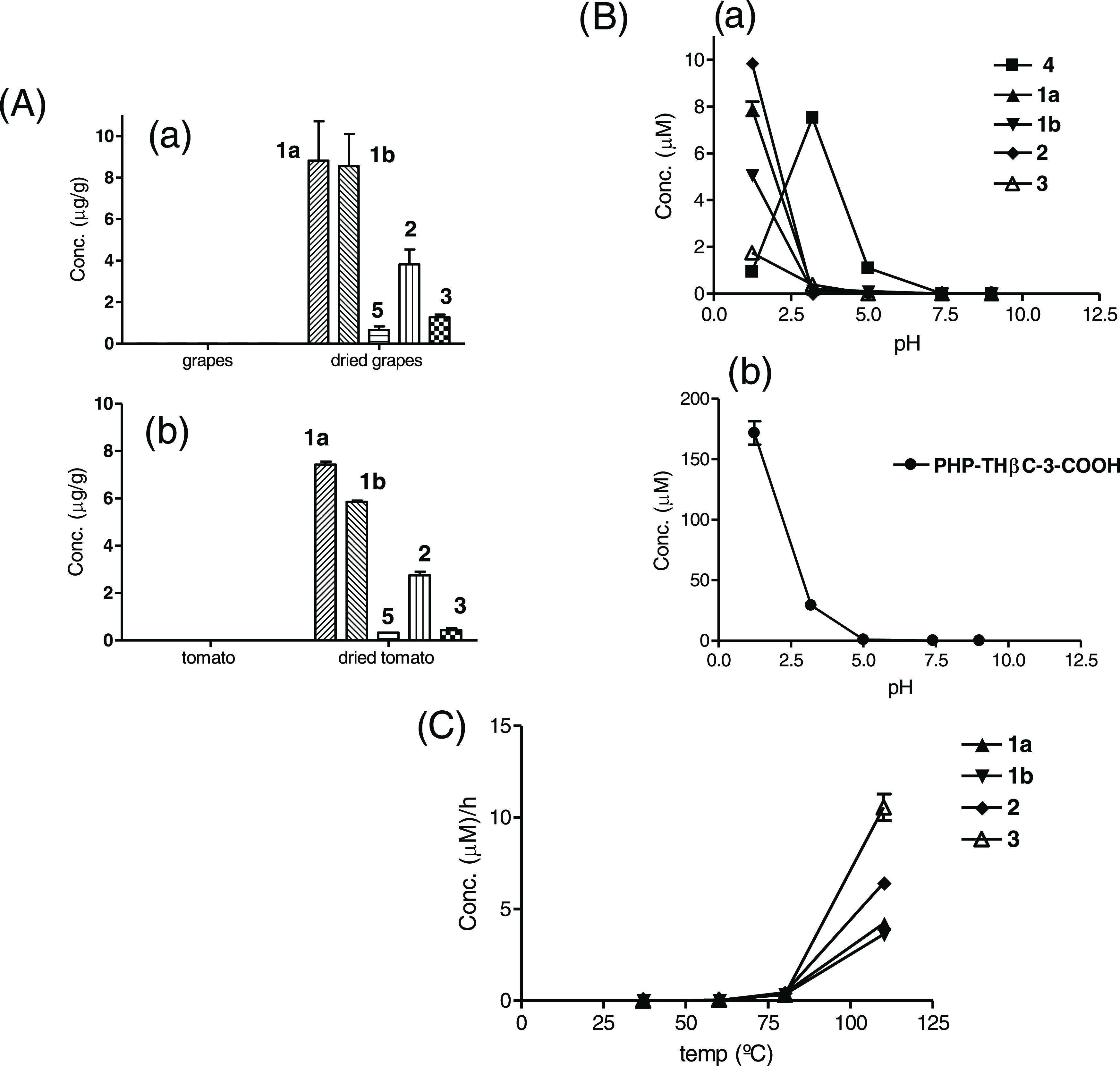
(A) Formation of carbohydrate βCs in grapes (a) and tomatoes (b) samples dried in an oven at 80 °C compared with raw samples. (B) Formation of carbohydrate βCs **1–4** (a) and PHP-THβC-3-COOH (b) in model reactions of tryptophan (0.5 g/L) and glucose (5 g/L) at various pHs (90 °C, 20 h). (C) Formation of carbohydrate βCs **1–3** with temperature and time (h) in model reactions of tryptophan (2 g/L) and glucose (40 g/L) at pH 3.1. Results are average of duplicates.

**Figure 6:**
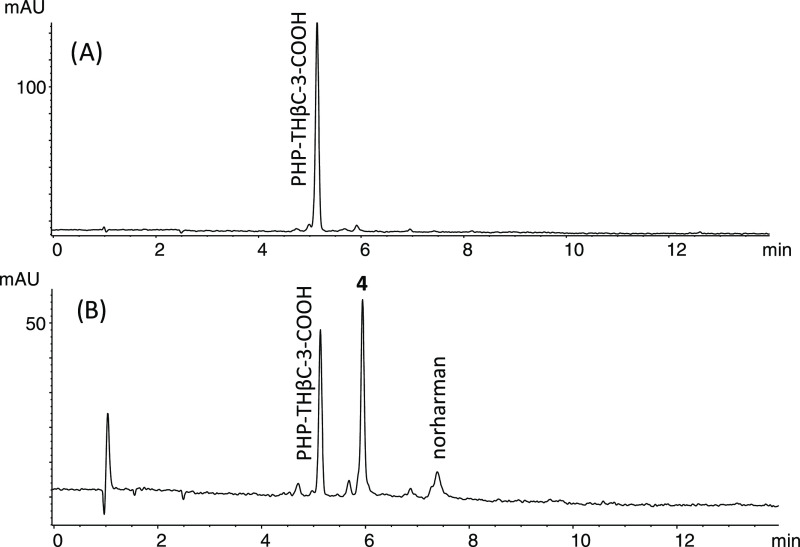
RP-HPLC chromatograms (A 254 nm) of PHP-THβC-3-COOH (200 μM, pH 5) before (A) and after heating at 140 °C for 4 h (B). The compound was redissolved when dried during heating.

The content of carbohydrate βCs **1–3** (Table 1) was high in processed foods and low in fresh or little processed foods, suggesting that these βCs formed during food processing (e.g., tomato puree vs fried tomato sauce or tomato juices made not from concentrate vs those made from concentrate). This fact was confirmed further in the laboratory as the carbohydrate βCs were formed during the heating and drying process of grapes and tomatoes (Figure 5). As shown in model reactions, compounds **1–3** can occur from a reaction of tryptophan with glucose (Figure 5). This formation highly increased under acidic conditions and with increasing temperature. It needed high temperatures as no formation of carbohydrate βCs **1–3** occurred at room temperature or under physiological conditions (37 °C), whereas low or trace amounts appeared at 60 °C (e.g., in the reaction of 2 g/L tryptophan and 40 g/L glucose, pH 3.1, 20 h). However, the formation of these compounds occurred when heating at 80 °C or higher temperatures. Remarkably, the relative proportions of compounds **1–3** changed with temperature, and **2** and **3** were favored over compounds **1a/b** at high temperatures (100 °C and above) (Figure 5).

l-Tryptophan reacts with glucose affording PHP-THβC-3-COOH (Figure 1).^12^ This THβC-3-COOH occurred in foods such as processed tomato products, fruit juices, and jams, and increased under acidic conditions and with increasing temperature.^12^ Here, PHP-THβC-3-COOH appeared in model reactions of tryptophan and glucose in higher amounts than the carbohydrate βCs **1–3** (Figure 5). PHP-THβC-3-COOH might be a possible precursor of the aromatic carbohydrate βCs **1–3** by oxidative decarboxylation and dehydration.^16^ However, no appreciable formation of the carbohydrate βCs **1–3** occurred when PHP-THβC-3-COOH was heated at a high temperature (e.g., 110–140 °C) or under oxidative conditions (H_2_O_2_ and peroxidase). Instead, it afforded the corresponding oxidation and decarboxylation product 1-(1,2,3,4,5-pentahydroxypent-1-yl)-β-carboline (**4**) and norharman (Figure 6). Indeed, βC **4** appeared in model reactions of tryptophan and glucose at acidic pH along with PHP-THβC-3-COOH (Figure 5B). No formation of βCs **1-3** resulted from **4** during heating at high temperatures (110–140 °C), and neither compound **2** or **3** resulted from **1** under the same conditions. Then, compounds **1–3** formed in a distinct way to PHP-THβC-3-COOH and **4**. A free carboxylic group was required because the reaction to give **1–3** occurred with tryptophan, but tryptamine did not afford the compounds when reacted with glucose at high temperatures and under acidic conditions, whereas tryptophan methyl ester afforded only trace amounts likely due to ester hydrolysis.

**Figure 7:**
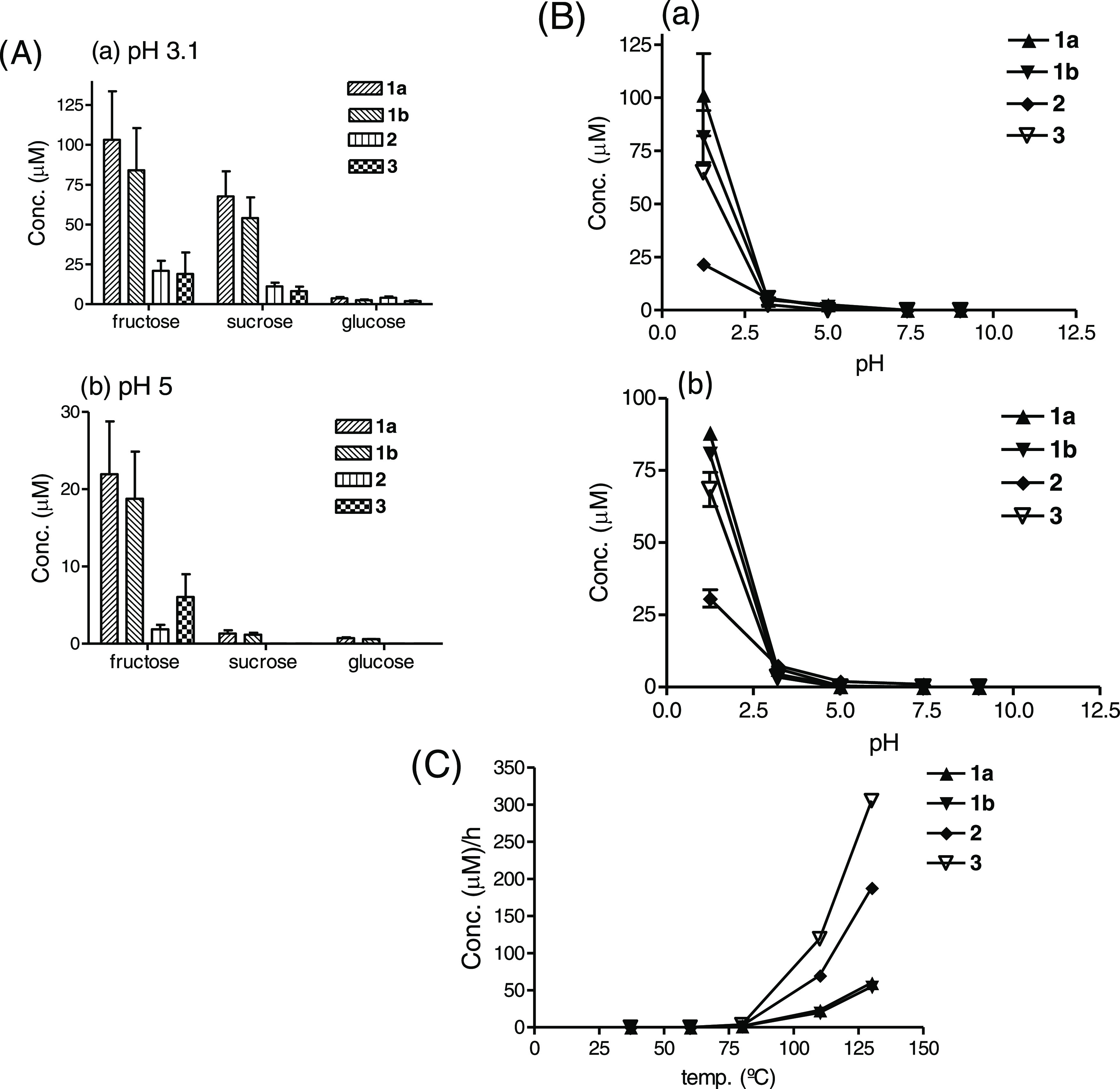
(A) Formation of carbohydrate βCs **1–3** in model reactions of tryptophan (2 g/L) and glucose (40 g/L), sucrose (69.1 g/L), or fructose (36.6 g/L) at pH 3.1 (a) or pH 5 (b) (20 h, 80 °C). (B) Formation of carbohydrate βCs **1–3** in model reactions of tryptophan (0.5 g/L) and fructose (4.5 g/L) (a) or sucrose (8.5 g/L) (b) at various pHs (20 h, 90 °C). (C) Formation of carbohydrate βCs **1–3** with temperature and time (h) in model reactions of tryptophan (2 g/L) and fructose (36.6 g/L) at pH 3.1. Results are average of duplicates.

**Figure 8:**
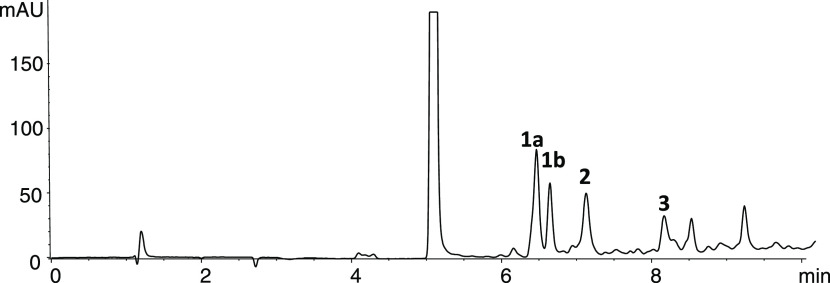
HPLC chromatogram (254 nm) of carbohydrate-derived βCs formed in model reaction of 3-deoxyglucosone (0.1 mg/mL) and tryptophan (0.5 mg/mL) at pH 3 (110 °C, 2h) (B). Compounds are as in Figure 1.

Interestingly, the carbohydrate βCs **1–3** were formed when tryptophan reacted with fructose or sucrose under acidic conditions and heating. Under the same conditions, the formation of βCs **1–3** from fructose or sucrose was much higher than from glucose (Figure 7A and also Figures 7B and 5B). The reaction was also highly favored in acidic pH although the formation of **1–3** from fructose occurred even in up to pH 5 to a low extent (Figure 7). It increased with temperature, and high temperatures favored **2** and **3** over **1** (Figure 7C). Under acidic conditions (pH 1–3) and heating, the formation of **1–3** from sucrose was close to fructose; however, it was much lower at pH 5 (Figure 7). Compared with sucrose, other disaccharides such as maltose or lactose gave very low levels or trace amounts of **1–3** under the same conditions (pH 3.1, 90 °C, 20 h). In model reactions of tryptophan and fructose, compound **6** (1-(1-keto-3,4,5-trihydroxypent-1-yl)-THβC-3-COOH) was detected (Figure 3). This compound was isolated by collecting the corresponding peak at the exit of the HPLC column, and after heating (110 °C, 3 h) or oxidation with 0.025 mg/mL HRP, 500 μM H_2_O_2_ (40 min, 37 °C) gave the corresponding aromatic βC (*m*/*z* at 301 (MH^+^) and 211 MH^+^-C_3_H_6_O_3_) but not **1–3**. The presence of this THβC could indicate a possible involvement of 3-deoxyglucosone in the reaction. Indeed, when authentic 3-deoxyglucosone (0.1 mg/mL) was reacted with tryptophan (0.5 mg/mL) under acidic conditions (pH 1.3 and 3.1) at 90 °C and 110 °C, the carbohydrate βCs **1–3** were formed (Figure 8). The concentrations produced (110 °C and pH 3.1, 2 h) were 27.2 ± 0.42 μM (**1a**), 22.8 ± 1.8 μM (**1b**), 24.5 ± 0.5 μM (**2**), and 14.0 ± 0.7 μM (**3**).

### Activity of the Carbohydrate-Derived βCs 1–4 as MAO Inhibitors, Antioxidants, and DNA-Interaction Agents

**Figure 9:**
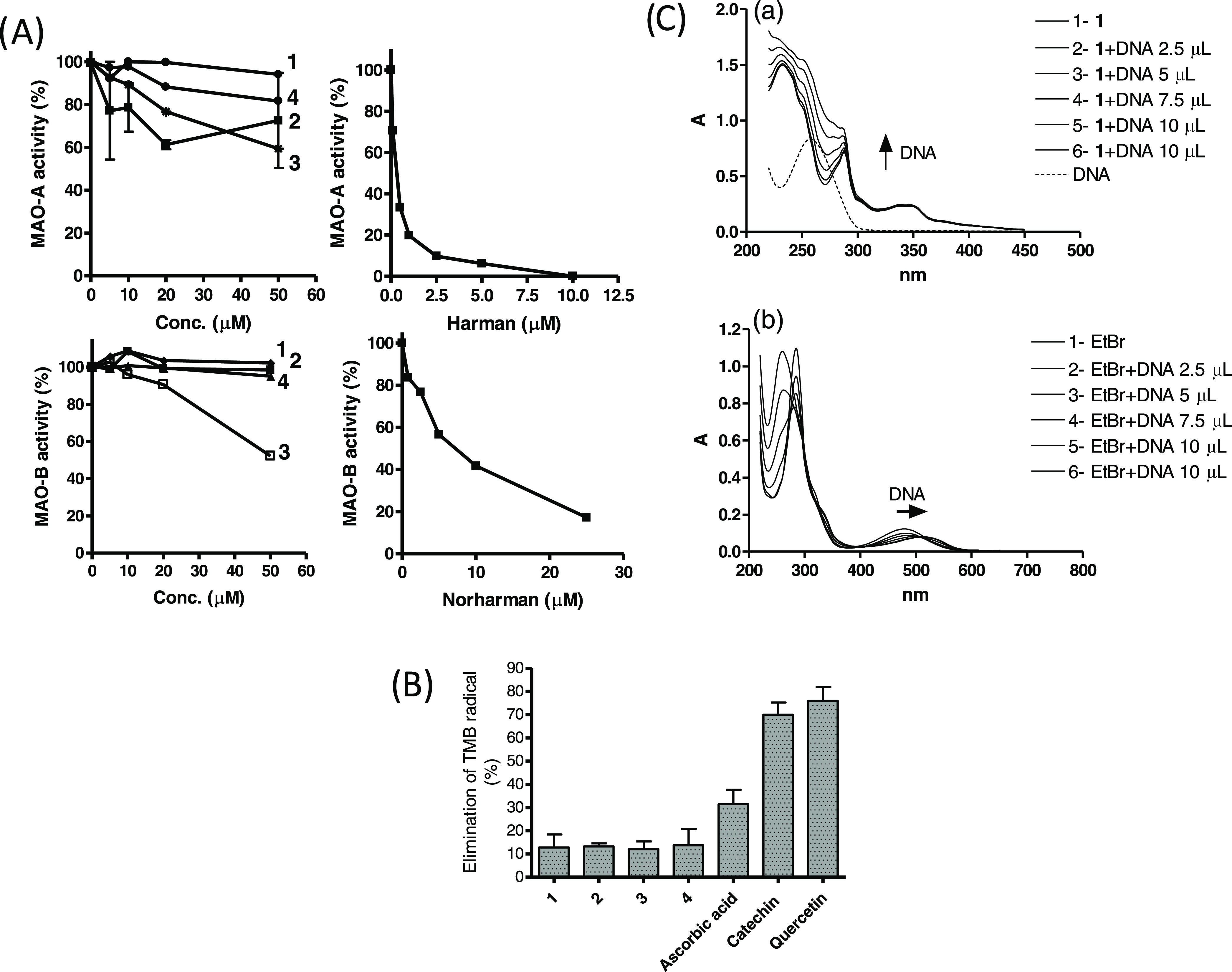
(A) Inhibition of human MAO-A and -B by carbohydrate βCs **1-4**, harman, and norharman. (B) Elimination of the cation radical TMB^+•^ in the presence of carbohydrate βCs **1–4** (30 μM) and antioxidants (30 μM). (C) Interaction of carbohydrate βC **1** (a) and ethidium bromide (b) with calf thymus DNA measured with UV–vis spectra. Increasing concentrations of DNA are added to a solution of **1** (25 μM) or ethidium bromide (25 μM). A mixture of isomers of **1** is used.

Inhibition of MAO, antioxidant effects, and interaction with DNA could be expected as biological activities of the carbohydrate-derived βCs. The carbohydrate βCs **1–4** were studied as inhibitors of human MAO-A and -B and none of the compounds significantly inhibited MAO compared to the βCs harman and norharman, which were good inhibitors (Figure 9A).^5,27^ The antioxidant activity of **1–4** was studied by scavenging of TMB^+•^ cation radical. The carbohydrate βCs had almost no antioxidant activity compared with ascorbic acid, catechin, or quercetin (Figure 9B). The βCs have been previously reported to interact with DNA.^32^ The interaction of βCs **1–4** with DNA was studied by spectrophotometry UV–vis following standard procedures.^31^ None of the compounds showed a relevant interaction or intercalation with calf thymus DNA evidenced by changes in UV–vis spectra compared with DNA and compounds alone (Figure 9C). In the same experiment, ethidium bromide, a well-known DNA intercalator, interacted with DNA as shown by hypochromicity and the change in the maximum of the absorption band (bathochromicity) from 480 to 510–520 nm (Figure 9C).^29^

## Discussion

The carbohydrate-derived βCs were identified and quantified in foods. The βCs **1–3** occurred in many commercial foods, and the compounds **4** and **5** were identified as minor compounds. The βCs **1–3** occurred in higher amounts in processed foods, particularly those subjected to heating processes. They formed in a reaction of l-tryptophan with glucose at a high temperature and acidic pH. Noticeably, the βCs **1–3** also formed from other carbohydrates such as fructose and sucrose (Figures 2 and 7), and under the same conditions (e.g., molar concentration, reaction time, pH, and temperature), the formation of the βCs **1–3** from fructose occurred at a much higher rate than from glucose (Figures 5 and 7). The formation of **1–3** from fructose and sucrose also increased with the temperature and acidic pH. Fructose reacted with tryptophan to give **1–3**, whereas sucrose hydrolyzed under acidic pH and heating releasing fructose that reacted with tryptophan to give the compounds. No significant reaction was observed from tryptophan and carbohydrates under physiological conditions (37 °C and pH 7.4). The results show that foods containing tryptophan and carbohydrates subjected to processing conditions including heating or cooking will afford the carbohydrate βCs **1–3**. The results obtained indicate that fructose and sucrose (when hydrolyzed to fructose) should be the main carbohydrate precursors of the carbohydrate βCs **1–3** in foods. Fructose and sucrose are naturally present in foods and/or are added during food production or cooking (e.g., high fructose corn syrup, HFCS). Many foods are naturally rich in fructose such as fruits, dried fruits, and fruit juices. Some foods are also added with fructose and processed by heating such as fried tomato sauce, salad dressings, snack foods, baked foods, fast foods, breakfast cereals, cereal bars, and jams. Some of these foods contained relatively high levels of **1–3**. Sucrose is naturally present in foods, and it is also added during processing (heating) in foods such as fried tomato sauce and ketchup. Then, fructose or sucrose addition to processed and cooked foods could increase the carbohydrate-derived βC **1–3** in foods containing tryptophan. The βC **1** has been isolated from the juice of ripe fruits of *N. tangutorum* (desert cherry), and called tangutorids E (**1a**) and F (**1b**).^22^ Other βCs arising from highly processing (dehydrating) conditions such as flazin and perlolidin (furfural derivatives of βCs) were also identified in those fruits.^22^ However, as shown here, the βCs **1–3** occurred in many foods including dried fruits (Table 1). Therefore, the classical nomenclature initially used for carbohydrate βCs^17^ was preferred here because these compounds are mainly formed during processing/heating and usually do not appear in fresh fruits. Without more data on the biogenesis and quantitative levels of **1a/b** in *Nitraria* fruits, it can be expected that these βCs could have formed during ripening, drying, and processing of fruits.

**Figure 10:**
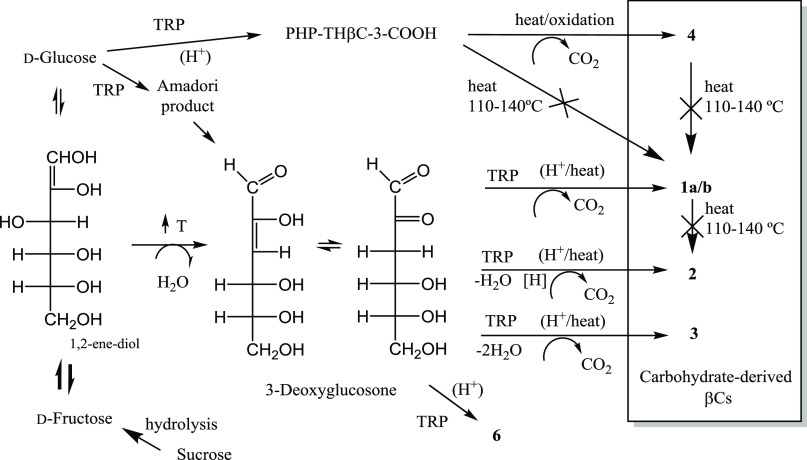
Formation of carbohydrate-derived βCs in foods. Glucose reacts with tryptophan affording the compound PHP-THβC-3-COOH that can be oxidized to the βC **4**. On the other hand, fructose (or sucrose after hydrolysis) or glucose affords 3-deoxyglucosone intermediate, which reacts with tryptophan to give the carbohydrate-derived βCs **1–3**. Compounds are as in Figure 1.

The reaction of tryptophan to give **1–3** occurred with both fructose and glucose, although with the former in a much higher rate. Glucose and fructose isomerize each other via 1,2-enediol. This fact could explain the same reaction occurring with both carbohydrates to give **1–3** (Figure 10). The reactive form of the sugar should be in the open chain (acyclic form). At room temperature and neutral pH, the acyclic form of fructose is about 0.5%, but it may increase up to 13.1% at 80 °C.^33^ Glucose is less than 0.04% in its acyclic form.^34^ Then, fructose exists in its acyclic form to a greater extent than glucose, which favors a higher reaction of this sugar. The initial stages of the Maillard reaction occur more rapidly with fructose,^35^ and fructose is more prone to dehydration. It dehydrates to give 3-deoxyaldoketose (3-deoxyglucosone or 3-deoxy-erythro-hexos-2-ulose) by the loss of one molecule of water from its 1,2-enediol (Figure 10).^36^ 3-Deoxyglucosone is an intermediate from the dehydration of fructose and glucose, and it is also formed from the Schiff base and Amadori product during glycation. It is present in foods^37−39^ and blood,^40^ and it is a precursor of advanced glycation end products (AGEs), and a marker of diabetes.^36,41,42^ The formation of 3-deoxyglucosone enables a further reaction with tryptophan to give the carbohydrate-derived βCs (Figure 10).

**Figure 11:**
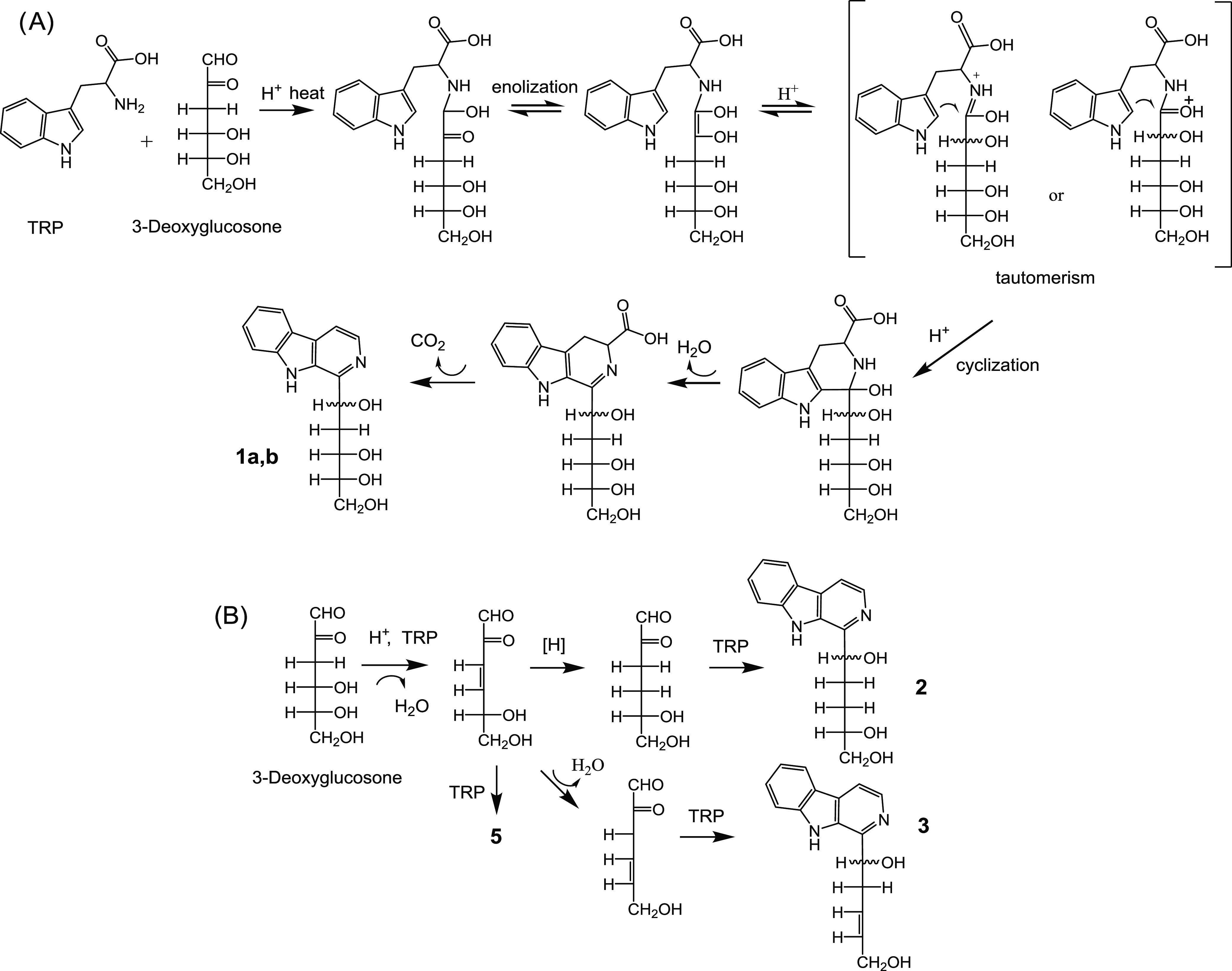
(A) Proposed mechanism for the formation of the carbohydrate-derived βCs **1a/b** in foods following reaction of tryptophan with the 3-deoxyglucosone intermediate formed from sugars. (B) Proposed mechanism for the formation of the βCs **2** and **3** following dehydration of 3-deoxyglucosone and reduction (βC **2**), or further dehydration (βC **3**) before cyclization to give the βCs. The βC **5** might come similarly from dehydration of 3-deoxyglucosone. The reaction of cyclization with tryptophan to give the βCs occurs as for **1a/b**.

PHP-THβC-3-COOH is the carbohydrate-derived tetrahydro-β-carboline (THβC) arising from the reaction of glucose and tryptophan (Figure 1).^12^ In model reactions, it occurred in higher amounts than the βCs **1–3** (Figure 5). This THβC increased with temperature and acidic conditions, and it appeared in foodstuffs with the highest content in tomato processed products, fruit juices, and jams.^12^ Here, the same foods contained high levels of the carbohydrate βCs **1–3** (Table 1). THβC-3-COOHs are direct precursors of aromatic βCs (e.g., norharman and harman) in foods by chemical or enzyme-catalyzed oxidative decarboxylation.^13,43^ The compound PHP-THβC-3-COOH has been mentioned as a possible precursor of the carbohydrate βCs **1–3**.^16^ However, the results in this work rule out this route. Upon heating or oxidation, PHP-THβC-3-COOH gave the carbohydrate βC **4** and norharman but not **1–3**. This agrees with the fact that polyols do not dehydrate easily. Then, PHP-THβC-3-COOH and **4** occur by a different way from βCs **1–3**. In model reactions of tryptophan and glucose, very low amounts or no βCs **1–3** occurred at moderate temperatures (60**–**70 °C or lower temperatures), whereas PHP-THβC-3-COOH was favored (Figure 5).^12^ In contrast, **1–3** occurred at higher temperatures. The βC **4** was accompanied with the presence of PHP-THβC-3-COOH; however, the βCs **1–3** were not accompanied with any detectable presence of their corresponding THβC-3-COOHs by HPLC-MS. The formation of **1–3** by a distinct way to **4** also explains the different concentrations among these compounds in foods. A scheme for the formation of these compounds is in Figure 10. A Pictet–Spengler condensation between tryptophan and glucose gives PHP-THβC-3-COOH that may suffer oxidative decarboxylation to give **4**. On the other hand, the βCs 1-3 arise from fructose or glucose that isomerize through 1,2-enediol and gives by dehydration 3-deoxyglucosone (also produced through the Amadori product of glucose). As shown here, 3-deoxyglucosone reacts with tryptophan to give the βCs **1**–**3**. The mechanism to give these compounds includes a reaction of cyclization and oxidative decarboxylation that is accompanied with the loss of the carbonyl group at position C-2 of 3-deoxyglucosone, which is converted to OH. Figure 11A shows a mechanism proposed for this reaction to give the βC **1a/b** based on keto–enediol or enamine–imine tautomerism. The compounds **2** and **3** could form in the same way as **1**, but the reactions accompanied with further dehydration from 3-deoxyglucosone before cyclization to the βC. Thus, the βC **2** could come from the loss of water from 3-deoxyglucosone followed by a reduction of the enone group, that is susceptible to reduction,^44^ and the βC **3** by an additional loss of water (Figure 11B). The βC **5** could arise in the same way following a loss of water from 3-deoxyglucosone. The formation of **2** and **3** can occur simultaneously to **1** as no significant formation of these compounds occurred from **1** when heated (Figure 10) probably due to the influence of the carbonyl in C-2 of 3-deoxyglucosone in the reaction of dehydration.

Many commercial foods contained the carbohydrate βCs **1**–**3** ranging from undetectable to several μg/g or mg/L levels (Table 1). Like norharman and harman,^1,4,13,27^ the βCs **1**–**3** are aromatic βCs, but data about these compounds in foods are scarce^1,17,20^ The highest content of **1**–**3** was found in processed tomato products and sauces, in agreement with previous results.^17,20^ They were also found in relatively high amounts in dehydrated fruits, fruit and tomato juices, jams, breads, cookies, chocolates, and breakfast cereals. Processed and heated foods contained higher amounts than fresh or unprocessed foods (Table 1). This was evidenced in low processing tomato products (e.g., tomato puree) compared with high processing products (e.g., fried tomato sauce or ketchup) and in dried vs fresh fruits. The presence of **1**–**3** in processed fruits and vegetables could be favored owing to the relatively high content of fructose in those foods. Foods containing **1**–**3** may also contain norharman and harman.^14^ The βCs **1**–**3** were found in dehydrated fruits, particularly raisins, which also contain norharman and harman in high amount.^4^ Fermented beverages such as wines and vinegars did not contain the βCs **1**–**3** but contain norharman and harman.^1,13^ An exception was balsamic vinegar where formation arises from heat-concentrated must.^17^ The carbohydrate βCs **1**–**3** were not significantly found in coffee or tea but appeared in chocolate. In contrast, coffee is one of the highest dietary sources of norharman and harman.^13,27^ Compared to norharman and harman, a higher amount of **1**–**3** was found in processed tomato, fruit and vegetable juices, cereals, and cookies and lower amount in coffee, wines, alcoholic beverages, or vinegars (except balsamic vinegar). The exposure to norharman, harman, and the βCs **1**–**3** together was estimated to reach levels of mg/person/day.^1,13^ A more accurate estimation of 0.93 μg/kg body weight/day has been reported for the uptake of **1–2** based on tables of food consumption.^20^ The results in this work agree with previous estimations and also suggest that the daily uptake of **1**–**3** may increase during cooking of foods containing tryptophan and sugars. Although the pattern of carbohydrate βCs differed among foods, usually **1a/b** were the main compounds (Table 1). At high temperatures (100 °C and above), the formation of compounds **2–3** increased. Then, very high temperatures during elaboration or cooking could produce more of the βCs **2**–**3** resulting from higher dehydration. The formation of **1**–**3** mainly depends on tryptophan that is the limiting factor and also on carbohydrates, particularly fructose as the main contributing carbohydrate. A control of these precursors avoiding high temperatures during food processing or cooking, particularly under acidic conditions, could reduce the levels of these carbohydrate βCs.

The βCs are bioactive compounds with a wide range of biological activities including interaction with CNS receptors, enzyme inhibition (MAO, kinases), anticancer, antimicrobial, and antioxidant actions. They are co-mutagenic in the presence of aromatic amines and could be bioactivated to give neurotoxic *N*-methyl-β-carbolinium cations.^3^ The βCs exert their effects into CNS through interaction with MAO enzymes and brain receptors.^1,3^ Thus, the βCs norharman and harman isolated from foods are inhibitors of MAO.^4,27^ However, as shown here, the carbohydrate βCs **1**–**4** did not inhibit MAO significantly, and therefore these compounds do not contribute to MAO inhibition in contrast to norharman and harman. THβCs and βCs exhibit antioxidant effects and react with hydroxyl radicals.^1,11^ However, compounds **1**–**4** had poor antioxidant activity compared with ascorbic acid or phenolic compounds (quercetin and catechin). This agrees with the relatively low antioxidant activity reported for **1a/b** and with the fact that aromatic βCs have low antioxidant activity compared to THβCs^22,45^ On the other hand, it has been reported that the aromatic βCs could interact with DNA.^10,32^ Results of this work suggest that the carbohydrate βCs **1–4** have no relevant activity of direct interaction with DNA. However, as the compounds with a β-carboline residue are bioactive substances and their activity depends on the specific structure of β-carboline,^1^ it is likely that the carbohydrate-derived βCs have other activities and act on different targets.

The carbohydrate-derived βCs **1**–**5** were identified and quantified in commercial foods. Compounds **1–3** appeared in a range from undetectable level to 11.4 μg/g, with **1a/b** being the major compounds. Many foods, especially those processed by heating and cooking contained carbohydrate-derived βCs, confirming a daily exposure to these compounds in the diet. The highest content of **1–3** was found in processed tomato products such as fried tomato sauce, tomato juices, ketchups, and dried tomato. Also, relatively high concentrations were found in sauces, processed fruits such as dried fruits and juices, and baked foods such as toasted breads, biscuits, and chocolate. Processed foods contained higher amounts than fresh or unprocessed foods. The formation of **1–3** occurred during heating as shown in dried tomato and raisins. High temperatures favored **2** and **3** over **1a/b**. The formation of **1–3** occurred from a reaction of tryptophan with glucose at temperatures higher than 80 °C under acidic conditions. Remarkably, results in this study show that the βCs **1–3** also occurred from a reaction of tryptophan with fructose or sucrose. The formation of **1–3** from fructose was much higher than from glucose, suggesting that fructose is the main carbohydrate involved. Sucrose formed the βCs **1–3** after acid hydrolysis. It is demonstrated for the first time that the mechanism of formation of the carbohydrate βCs **1–3** occurs in a new route that involves the reaction of tryptophan with 3-deoxyglucosone arising from fructose or glucose. A mechanism of reaction is proposed for this reaction that involves the keto–enediol or enamine–imine tautomerisms. In contrast, the βC **4** comes from the oxidative decarboxylation of PHP-THβC-3-COOH. The carbohydrate-derived βCs **1–4** did not inhibit MAO-A or B, were poor antioxidants, and had no remarkable activity of interaction or intercalation with DNA.
